# Effect of Previous COVID-19 Vaccination on Humoral Immunity 3 Months after SARS-CoV-2 Omicron Infection and Booster Effect of a Fourth COVID-19 Vaccination 2 Months after SARS-CoV-2 Omicron Infection

**DOI:** 10.3390/v14112458

**Published:** 2022-11-06

**Authors:** Jinsoo Kim, Hyeonji Seo, Han-Wool Kim, Dongbum Kim, Hyung-Joo Kwon, Yong-Kyun Kim

**Affiliations:** 1 Department of Microbiology, Hallym University College of Medicine, Chuncheon 24252, Korea; 2 Division of Infectious Diseases, Department of Internal Medicine, Hallym University Sacred Heart Hospital, Hallym University College of Medicine, Anyang 14068, Korea; 3 Department of Pediatrics, Hallym University Sacred Heart Hospital, Hallym University College of Medicine, Anyang 14068, Korea; 4 Institute of Medical Science, Hallym University College of Medicine, Chuncheon 24252, Korea

**Keywords:** COVID-19 vaccine, SARS-CoV-2 Omicron variant infection, optimal timing of vaccination, fourth dose

## Abstract

In this study, we aimed to determine the effect of COVID-19 vaccination on 3-month immune response and durability after natural infection by the Omicron variant and to assess the immune response to a fourth dose of COVID-19 vaccination in patients with prior natural infection with the Omicron variant. Overall, 86 patients aged ≥60 years with different vaccination histories and 39 health care workers (HCWs) vaccinated thrice before Omicron infection were enrolled. The sVNT50 titer was significantly lower in patients with incomplete vaccination before SARS-CoV-2 infection with the S clade (*p* < 0.001), Delta variant (*p* < 0.001), or Omicron variant (*p* = 0.003) than in those vaccinated thrice. The sVNT results against the Omicron variant did not differ significantly in patients aged ≥60 years (*p* = 0.49) and HCWs (*p* = 0.17), regardless of the recipient receiving the fourth dose 2 months after COVID-19. Incomplete COVID-19 vaccination before Omicron infection for individuals aged ≥60 years conferred limited protection against homologous and heterologous virus strains, whereas two or three doses of the vaccine provided cross-variant humoral immunity against Omicron infection for at least 3 months. However, a fourth dose 2 months after Omicron infection did not enhance immunity against the homologous strain. A future strategy using the bivalent Omicron-containing booster vaccine with appropriate timing will be crucial.

## 1. Introduction

The COVID-19 pandemic is ongoing, sustained by the emergence of new variants of concern, such as Omicron (B.1.1.529) and its subvariants [1]. A higher rate of reinfection associated with immune escape has been reported with the Omicron variant than with earlier variants [2,3]; previous infections with earlier Omicron subvariants (BA.1 or BA.2) do not appear to generate robust immunity against newer subvariants such as BA.4 and BA.5 [4,5,6].

Reinfection with SARS-CoV-2 may increase the risk of adverse health outcomes [7] and can increase the public health burden and result in post-COVID-19 conditions [7,8,9]. Reinfection in populations with a high risk of severe COVID-19 outcomes, such as older adults, may lead to COVID-19 becoming endemic [10,11]. Therefore, preventing reinfection is important. The optimal and rational use of existing vaccines, considering their effectiveness during previous waves, is a key component [12,13]. Moreover, owing to a substantial increase in people who contracted SARS-CoV-2 during recent Omicron surges, an optimal vaccination strategy is needed to maximize the effect of hybrid immunity provided by the combination of prior natural infection and vaccination, to optimize protection against reinfection [14,15,16,17].

However, the guidelines regarding vaccination of people with prior SARS-CoV-2 infection vary considerably in the timing after recovery [18,19,20,21]. At present, the optimal timing of vaccination after recovery from SARS-CoV-2 infection is difficult to determine because of limited data on the duration of immunity after natural infection by the Omicron variant. In addition, although the timing of vaccination after recovery applies to the receipt of primary-series or booster doses in guidelines [18], it has not been determined whether the optimal vaccination strategy after recovery differs according to vaccination history before SARS-CoV-2 infection.

We evaluated whether the 3-month immune response and durability after natural infection by the Omicron variant is affected by COVID-19 vaccination received before infection, to provide evidence on which to base timing of vaccination of individuals with prior SARS-CoV-2 infection. In addition, we assessed the immune response to a fourth dose of COVID-19 vaccine in patients with prior natural infection with the Omicron variant.

## 2. Materials and Methods

### 2.1. Study Population and Clinical Data Collection

This study was conducted at Hallym University Sacred Heart Hospital, an 829-bed university-affiliated hospital in Anyang, South Korea.

The study population included patients with SARS-CoV-2 infection confirmed on positive reverse-transcriptase polymerase chain reaction (RT-PCR) testing in February and March 2022 during an Omicron surge in South Korea. The specific pandemic wave underlying the present study was the fifth wave, predominantly caused by the Omicron variant that started on 30 January 2022, and BA.1 was the predominant strain between 1 January and 19 March 2022 [22,23]. The Korean government implemented at-home isolation for mild illness defined as various signs and symptoms of COVID-19 without dyspnea requiring oxygen therapy in October 2020, and patients requiring focused care, such as those aged ≥60 years and those with pre-existing medical conditions, received health monitoring twice a day [24].

Individuals considered for inclusion were patients aged ≥60 years who had SARS-CoV-2 infection between 17 February and 16 March 2022, and received at-home treatment from Hallym University Sacred Heart Hospital. Healthcare workers (HCWs) without any pre-existing medical conditions aged 22–40 years who had mild SARS-CoV-2 infection between 16 February and 11 March 2022 were also included in this study. Serum samples were collected from both patients and HCWs for SARS-CoV-2 surrogate virus neutralization tests (sVNTs) to detect the antibody approximately 3 months after their PCR-confirmed SARS-CoV-2 infection between 17 May and 9 June 2022.

The eligible population was divided into 5 groups: patients who received incomplete vaccination (0 or 1 dose) before COVID-19 (Cohort 1), patients who received 2 vaccinations before COVID-19 (Cohort 2), patients who received 3 vaccinations (or their first booster) before COVID-19 (Cohort 3), patients who received 4 vaccinations (or their second booster) after recovery from COVID-19 (Cohort 4), and HCWs who had 3 vaccinations before COVID-19 (Cohort 5). The documented vaccination records of the patients and HCWs enrolled in this study were obtained from computerized and integrated vaccination registration data of the Korea Disease Control and Prevention Agency (KDCA).

### 2.2. Surrogate sVNT

Human angiotensin-converting enzyme 2 (hACE2) protein (cat. no. Z03516) and recombinant receptor-binding domain (RBD) of the SARS-CoV-2 spike protein-horseradish peroxidase (HRP) fusion protein (RBD-HRP protein; cat. nos. Z03594 for S clade, Z03614 for the Delta variant, and Z03730 for the Omicron variant) were purchased from GenScript Korea (Seoul, Korea), and sVNTs were performed according to the manufacturer’s instructions. Briefly, the hACE2 protein (1 µg/mL) was coated onto a 96-well plate (cat. no. 439454) at 4 °C overnight and blocked with phosphate-buffered saline (PBS) containing 1% bovine serum albumin for 1 h. During the blocking step, 100 µL of positive (each RBD-HRP protein, 1 µg/mL) and negative (buffer only) samples were prepared in PBS supplemented with 0.1% Tween-20 (PBS-T). Then, 50 µL of two-fold serially diluted serum was mixed with 50 µL of RBD-HRP protein (2 µg/mL) in PBS-T and incubated at 20–25 °C. After 30 min of incubation, 100 µL of the mixture was added to an hACE2-coated 96-well plate and incubated for 15 min at 20–25 °C in the dark. After rinsing with PBS-T three times, tetramethylbenzidine (TMB) peroxidase substrate (Kirkegaard and Perry Laboratories, Gaithersburg, MD) was added to each well, and the absorbance of each well at 450 nm was measured using a Spectra Max 250 microplate reader (Molecular Devices, San Jose, CA, USA). The absorbance values were normalized to values of the PBS-coated well and the hACE2-coated well, as described previously [25]. The serum sVNT titer (sVNT50) was defined as the reciprocal value of the sample dilution, which showed a 50% reduction in signal at 450 nm.

### 2.3. Statistical Analysis

Student’s t-test or the Mann–Whitney U test was used to compare differences between continuous variables, and Pearson’s chi-square test or Fisher’s exact test was used to compare the categorical variables, as appropriate. All serum samples without a 50% reduction in signal at 1:40 dilution were considered “non-detectable” and were arbitrarily assigned a value of 1:20. Two-tailed *p* values < 0.05 indicated statistical significance. All statistical analyses were performed using Statistical Package for the Social Sciences version 27.0 (IBM Corp., Armonk, NY, USA).

## 3. Results

### 3.1. Enrollment and Baseline Characteristics

**Figure 1:**
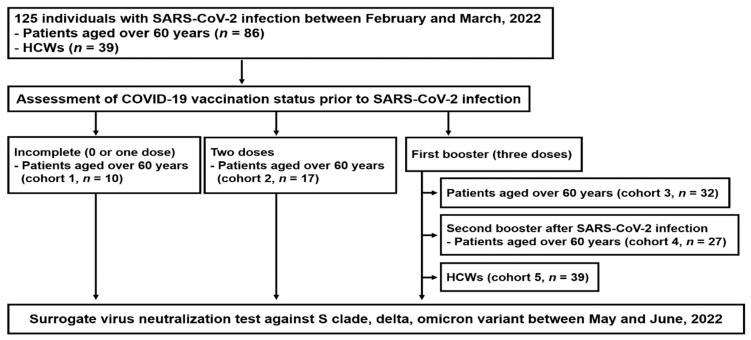
Study population.

Between 17 May 17 and 9 June 2022, 125 participants (86 patients aged ≥60 years and 39 HCWs) with primary PCR-confirmed SARS-CoV-2 infection between February 16 and March 16, 2022, presumed by the Omicron variant (BA.1), were enrolled in this study. We analyzed the results of the sVNT of serum samples obtained approximately 3 months after their infections in Cohorts 1–3 and 5, and approximately 4 weeks after the second booster (fourth dose) vaccination in Cohort 4 (Figure 1).

The demographics and COVID-19 vaccination records of the study cohort are summarized in Table 1. The median (interquartile range [IQR]) ages were 67.5 (62.8–68.3), 66.0 (63.0–68.5), 65.5 (63.0–67.0), and 65.0 (63.0–68.0) years in Cohorts 1, 2, 3, and 4, respectively. The median (IQR) age of the HCWs (Cohort 5) was 30.0 (27.0–37.0) years. In Cohort 1 (*n* = 10), one patient received one dose of the ChAdOx1-S vaccine before SARS-CoV-2 infection. In Cohort 2 (*n* = 17), 6 patients (35%) received 2 doses of the mRNA vaccine with a priming interval of 38 (IQR, 30–42) days and 11 patients (65%) received 2 doses of ChAdOx1-S with a priming interval of 77.0 (IQR, 77.0–77.0) days. In Cohorts 3 (*n* = 32) and 5 (*n* = 39), all patients received an mRNA vaccine as a booster dose with a median interval of 118.0 (IQR, 107.0–130.5) days and 175.0 (IQR, 169.0–192.0) days after the second dose, respectively. The proportion of heterologous boosters was 88% (28/32) and 85% (33/39) in Cohorts 3 and 5, respectively. In Cohort 4 (*n* = 27), 24 patients (24/27, 89%) received the mRNA vaccine and 3 patients (3/27, 11%) received the Nuvaxovid vaccine as a second booster, a median of 137.0 (IQR, 133.0–150.3) days and 137.0 (IQR, 133.0–149.5) days after the third dose, respectively. The median sampling intervals (IQR) from SARS-CoV-2 infection were 87.5 (85.5–93.5), 94.0 (87.5–99.0), 84.0 (83.0–87.8), 86.0 (84.0–90.0), and 81.0 (78.0–84.0) days in Cohorts 1, 2, 3, 4, and 5, respectively. In Cohort 4, the median interval between SARS-CoV-2 infection and the second booster vaccination was 60.0 (IQR, 55.0–67.0) days, and the median sampling interval from the second booster vaccination was 28.0 (IQR, 22.0–32.0) days.

### 3.2. Surrogate sVNT Results According to Vaccination Status before Omicron Variant Infection

**Figure 2:**
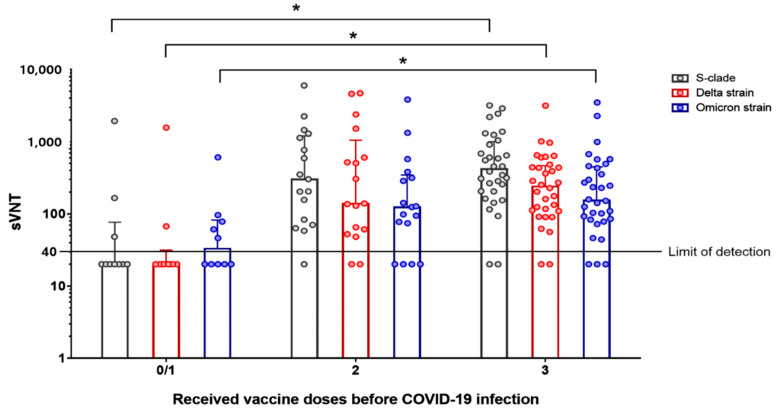
Surrogate SARS-CoV-2 virus neutralization test (sVNT) results according to vaccination status before Omicron variant infection. Black, red, and blue dots indicate the sVNT titer against the S clade, Delta variant, and Omicron variant, respectively. Bars indicate the medians and error bars indicate the interquartile ranges in each group. Asterisks indicate *p* < 0.05.

The sVNT results of enrolled patients aged ≥ 60 years according to vaccination status before SARS-CoV-2 infection are presented in Table 2 and Figure 2. Approximately 3 months after infection, the sVNT50 titer of Cohort 1 was significantly lower than that of Cohort 3 against the S clade (median, 20.0; IQR, 20.0–77.3 vs. median, 418.5; IQR, 204.5–1008.5; *p* < 0.001), Delta variant (median, 20.0; IQR, 20.0–31.8 vs. median, 239.5; IQR, 109.8–470.3; *p* < 0.001), and Omicron variant (median, 33.0; IQR, 20.0–82.5 vs. median, 154.5; IQR, 83.5–451.0; *p* = 0.003). In addition, five patients (5/10, 50%) in Cohort 1 showed detectable levels of antibody against the Omicron variant 3 months after infection, with lower amounts of detectable levels of antibody observed against the S clade type (30%) and Delta variant (20%). The sVNT50 titers of Cohort 2 against all three virus strains did not differ significantly from those of Cohort 3. Approximately 3 months after infection, Cohort 1 had low levels of sVNT50 titers against the S clade, Delta variant, and Omicron variant. In contrast, we detected efficient cross-variant immunity in serum samples from patients with Omicron variant infection who had been previously vaccinated twice (Cohort 2) or thrice (Cohort 3).

### 3.3. Surrogate sVNT Results after a Second Booster Vaccination (Fourth Dose) following Omicron Variant Infection

**Figure 3:**
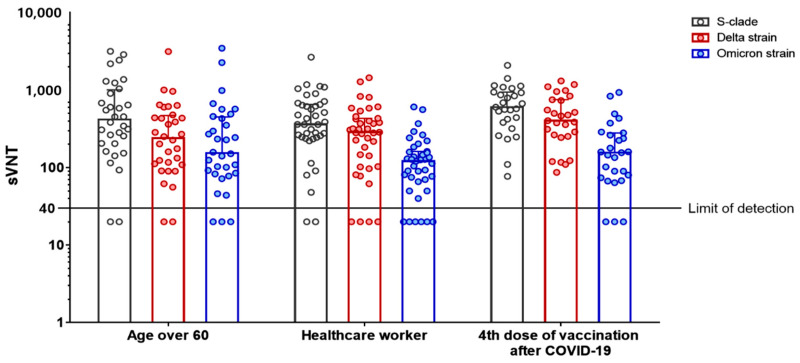
sVNT results after a second booster (fourth dose) vaccination after Omicron variant infection. Black, red, and blue dots indicate the sVNT titer against the S clade, Delta variant, and Omicron variant, respectively. Bars indicate the medians, and error bars indicate the interquartile ranges in each group. Asterisks indicate *p* < 0.05.

We compared the sVNT results after a second booster vaccination in patients aged ≥60 years (Cohort 4) with those of Cohorts 3 and 5 (Table 3 and Figure 3). About 3 months after infection, the sVNT50 titers in Cohort 3 were not significantly different from those of Cohort 4 against the S clade (median, 418.5; IQR, 204.5–1008.5 vs. median, 603.0; IQR, 383.0–945.0; *p* = 0.30) or Omicron variant (median, 154.5; IQR, 83.5–451.0 vs. median, 156.0; IQR, 74.0–281.0; *p* = 0.49), while those against the Delta variant were significantly lower in Cohort 3 (median, 239.5; IQR, 109.8–470.3 vs. median, 405.0; IQR, 253.0–753.0; *p* = 0.03). In addition, the sVNT50 titers of Cohort 5 against the Omicron variant did not differ significantly from those of Cohort 4 (median, 121.0; IQR, 66.0–162.0 vs. median, 156.0; IQR, 74.0–281.0; *p* = 0.17), while they were significantly lower in Cohort 4 against the S clade (median, 362.0; IQR, 246.0–652.0 vs. median, 603.0; IQR, 383.0–945.0; *p* = 0.03) and Delta variant (median, 287.0; IQR, 103.0–433.0 vs. median, 405.0; IQR, 253.0–753.0; *p* = 0.03). In contrast, the sVNT50 titers in Cohort 3 and Cohort 5 were not significantly different from each other against the S clade (*p* = 0.76), Delta variant (*p* = 0.64), and Omicron variant (*p* = 0.19), respectively. When we analyzed the cross-variant immunity of patients aged ≥60 years who received a second booster vaccination 2 months after Omicron variant infection in serum samples obtained 4 weeks after vaccination (Cohort 4), similar extents of immunity against Omicron were observed compared with cohorts vaccinated only three times regardless of age (Cohorts 3 and 5), with more efficient cross-variant immunity against the S clade and Delta variant.

## 4. Discussion

This study showed that patients who had not been previously vaccinated and were infected with the Omicron variant developed only limited levels of antibody against the S, Delta, and Omicron variants. Notably, only half of the patients with prior incomplete vaccination had detectable levels of antibody against the homologous strain 3 months after infection, with significantly lower titers than those of patients who had been fully vaccinated. In contrast, efficient cross-variant humoral immunity was maintained for 3 months in individuals who had been vaccinated twice or thrice before infection with the Omicron variant, both in patients aged ≥60 years and young HCWs. However, a second booster (fourth dose) 2 months after Omicron variant infection did not enhance immunity against the homologous strain.

Our study results showing differences in cross-variant immunity from Omicron variant infection according to prior COVID-19 vaccination status are consistent with those of several recent studies that evaluated cross-variant neutralization with or without vaccination [26,27,28,29]. However, compared with previous studies, our study was strengthened by the relatively larger number of convalescent serum samples obtained from patients with PCR-confirmed Omicron variant infection (*n* = 125), particularly with a longer interval of 3 months between infection and antibody evaluation. In addition, we observed the antibody response to a second booster vaccination using convalescent sera from patients aged ≥60 years who had been previously vaccinated thrice and infected with the Omicron variant.

Vaccination after recovery from SARS-CoV-2 infection is an important issue in planning future vaccination strategies because of the immune escape of newer variants. Although limited data are available regarding the risk of reinfection of unvaccinated individuals with past Omicron variant infection, one recent study revealed that reinfection can occur even in the early period after infection (<60 days), particularly in unvaccinated persons [30]. Thus, we posit that the results of our study can be used to inform the vaccination strategy in anticipation of further COVID-19 waves in the autumn and winter seasons. Although recent guidelines suggest COVID-19 vaccination for individuals with a documented history of SARS-CoV-2 infection, the recommended timing of vaccination varies considerably [18,19,20,21]. When we consider some degree of immunity to protect against reinfection by the natural infection of SARS-CoV-2 [31,32], it may be rational to delay vaccination until 3 months after infection [18,21,33]. However, our study revealed a lower degree of immunity against the homologous strain in patients with Omicron variant infection who had not received previous vaccination 3 months after infection, suggesting that the primary series might be performed with a shorter timeframe of less than 3 months, particularly during waves by circulating variants with increased risk of reinfection. Reinfections that occur in individuals who are already seropositive at baseline are associated with lower levels of neutralizing antibody [34]. Considered together, we advocate following the vaccination strategy recommended by the KDCA if at least 3 weeks have passed after SARS-CoV-2 infection in cases of prior incomplete vaccination [35].

One important finding of our study is that a second booster 2 months after Omicron variant infection did not further enhance immunity against the Omicron variant. This is consistent with recent studies that revealed limited peak boosting when high levels of humoral immunity existed before a fourth vaccination (ceiling effect) [36] and an extended time interval between infection and subsequent vaccination, resulting in a more improved immune response [33]. In addition, studies have shown that delaying the interval between doses is associated with a stronger antibody response [37,38], which suggests that the timing of the booster dose is associated with the magnitude of pre-existing immunity. We assume that the timing of a second booster or a fourth dose in a recent SARS-CoV-2 infection is an important issue to maximize the benefit of a fourth dose at this stage of the pandemic. Considering our results, an interval longer than 2 months between infection and the fourth dose should be considered. Further research is needed to explore the effect of vaccine type and varying host immunity on the optimal timing of a second booster in people with recent infection.

Another important finding of this study is that HCWs and patients aged ≥60 years had a similar level of humoral response, despite the different vaccine types and intervals between vaccine doses. We considered young HCWs representing healthy population as control group to patients aged ≥ 60 years, which may help determine whether to have different vaccination strategy according to ages. In addition, a fourth dose vaccination to HCWs is an important issue to maintain the function of the health care system during pandemic. The kinetics of humoral immunity to repeated COVID-19 vaccination in different age groups remain unclear, and few studies have used sera from HCWs who previously received a third dose and were infected with the Omicron variant. Our results suggest that HCWs have immune durability comparable to that of a third dose, and a second booster dose can be potentially effective in HCWs in a manner similar to that in older adults. Although there is a lack of consensus regarding administration of a second booster to HCWs because of the low vaccine efficacy against infections [39,40], these results can inform policymakers [40]. Further longitudinal data on the kinetics of antibody response over longer periods following natural infection with larger number of patients and HCWs is essential for optimal strategy.

Our study had some limitations. First, the sample size of this cohort was small. Second, we did not collect serum samples serially between vaccine doses or infection and vaccination in a well-designed manner. Third, we performed sVNTs using an enzyme-linked immunosorbent assay plate to evaluate the virus–host interaction rather than using conventional virus neutralization tests. Fourth, we did not include information on pre-existing pathological conditions of the study patients that might influence the antibody response to vaccinations. We found that there were no patients aged ≥ 60 years who received organ transplantation and rituximab as treatment regimen for autoimmune systemic disease, which can significantly affect the antibody response [41,42]. However, we posit that future studies should include information on comorbidities to support the optimal policy of vaccine administration in immunocompromised patients. Finally, both patients aged ≥ 60 years and HCWs enrolled in this study had mild illness. The antibody responses in patients who experienced severe disease may differ from those in subjects of this study, because the severity of clinical manifestations in COVID-19 patients are correlated with the level of titers [43,44] and the durability of SARS-CoV-2 antibodies [45,46]. In addition, we did not assess factors affecting immunogenicity among HCW, while smoking can be associated with humoral response [47,48]. Future studies should include thorough assessments of antibody response in recovered or naïve HCWs, because there is limited data available despite the important role within the pandemic and the occupational risk for exposure of HCWs. Despite these limitations, we believe that our study is valuable in providing evidence to recommend a more precise timing of vaccination for people with prior SARS-CoV-2 infection. Our study has important clinical implications for further well-designed research to evaluate whether the timing of COVID-19 vaccination after natural infection should be affected by any previous receipt of COVID-19 vaccination and the optimal timing of a second booster dose for individuals with prior natural infection. The bivalent omicron-containing vaccine can be considered as a second booster, as it was associated with superior immunogenicity and no evident safety concerns [49].

## 5. Conclusions

Our study shows that incomplete COVID-19 vaccination prior to Omicron variant infection in adults aged ≥60 years confers limited protection against homologous and heterologous strains, while efficient cross-variant humoral immunity might be maintained for 3 months in individuals who have been vaccinated twice or thrice before Omicron variant infection. However, a second booster vaccination (a fourth dose) 2 months after Omicron variant infection did not enhance immunity against the homologous strain. The timing of vaccination in a population with a history of SARS-CoV-2 infection should be determined according to the baseline immunity developed by vaccination before SARS-CoV-2 infection. As an integrated future perspectives, the bivalent omicron-containing booster vaccine should be considered priorly for elderly patients with incomplete COVID-19 vaccination before SARS-CoV-2 omicron infection, and for HCWs who have an important role within the pandemic and higher risk of occupational exposure.

## Figures and Tables

**Table 1:** Demographics of each cohort of patients according to their vaccination history before and after SARS-CoV-2 infection.

	Aged ≥ 60 Years				
Variables	Incomplete vaccination ^a^ (*n* = 10)	2nd Vaccination (*n* = 17)	3rd Vaccination (*n* = 32)	4th Vaccinationafter COVID-19 (*n* = 27)	Healthcare Worker (*n* = 39)
Age, years	67.5 (62.8–68.3)	66.0 (63.0–68.5)	65.5 (63.0–67.0)	65.0 (63.0–68.0)	30.0 (27.0–37.0)
Sex, male	4 (40.0)	7 (41.2)	17 (53.1)	17 (63.0)	10 (25.6)
Primary vaccination before SARS-CoV-2 infection					
Two doses of mRNA vaccine ^b^	NA	6 (35.3)	4 (12.5)	0	6 (15.4)
Interval between 1st and 2nd doses, days	NA	38.0 (30.0–42.0)	21.5 (21.0–31.8)	NA	24.5 (21.0–28.5)
Two doses of ChAdOx1-S	NA	11 (64.7)	25 (78.1)	26 (96.3)	30 (76.9)
Interval between 1st and 2nd doses, days	NA	77.0 (77.0–77.0)	77.0 (77.0–77.0)	77.0 (77.0–77.0)	84.0 (81.8–85.3)
Heterologous vaccination ^c^	NA	0	3 (9.4)	1 (3.7)	3 (7.7)
Interval between 1st and 2nd doses, days	NA	NA	77.0 (77.0–77.0)	77	77.0 (74.0–80.0)
3rd vaccination before SARS-CoV-2 infection					
mRNA vaccine ^d^	NA	NA	32 (100.0)	27 (100.0)	39 (100.0)
Interval between 2nd and 3rd doses, days	NA	NA	118.0 (107.0–130.5)	119.0 (105.0–121.0)	175.0 (169.0–192.0)
Homologous booster	NA	NA	4 (12.5)	0	6 (15.4)
Heterologous booster ^e^	NA	NA	28 (87.5)	27 (100.0)	33 (84.6)
4th vaccination after SARS-CoV-2 infection					
Time interval from SARS-CoV-2, days	NA	NA	NA	60.0 (55.0–67.0)	NA
mRNA vaccine ^f^	NA	NA	NA	24 (88.9)	NA
Interval between 3rd and 4th doses, days	NA	NA	NA	137.0 (130.0–150.3)	NA
Nuvaxovid vaccine	NA	NA	NA	3 (11.1)	NA
Interval between 3rd and 4th doses, days	NA	NA	NA	137.0 (130.0–149.5)	NA
Sampling interval, days					
From SARS-CoV-2 infection	87.5 (85.5–93.5)	94.0 (87.5–99.0)	84.0 (83.0–87.8)	86.0 (84.0–90.0)	81.0 (78.0–84.0)
From 4th vaccination	NA	NA	NA	28.0 (22.0–32.0)	NA

Data are presented as numbers (%) or medians (interquartile range) unless indicated otherwise. ^a^ No vaccination or 1^st^ vaccination before SARS-CoV-2 infection. One patient received one dose of the ChAdOx1-S vaccine before SARS-CoV-2 infection. ^b^ Three patients received two doses of mRNA-1273 vaccination and thirteen patients received two doses of BNT162b2 vaccination. ^c^ Seven patients had heterologous vaccination with ChAdOx1-S vaccination followed by BNT162b2 vaccination. ^d^ Thirty-eight patients had mRNA-1273 booster vaccination, and sixty patients had BNT162b2 booster vaccination. ^e^ Thirty-six patients received two doses of ChAdOx1-S vaccination followed by mRNA-1273 booster vaccination, and fifty-two patients received two doses of ChAdOx1-S vaccination followed by BNT162b2 booster vaccination. ^f^ Two patients had mRNA-1273 vaccination, and twenty-two patients had BNT162b2 vaccination. NA, not applicable.

**Table 2:** Comparison of serologic results in each cohort of patients aged ≥60 years according to their vaccination history before SARS-CoV-2 infection.

Variables	Incomplete Vaccination ^a^ (*n* = 10)	2nd Vaccination (*n* = 17)	3rd Vaccination (*n* = 32)	*p* Value ^b^	*p* Value ^c^
Serologic test					
S clade, sVNT50					
Detectable antibody titer ^d^	3 (30.0)	16 (94.1)	30 (93.8)	<0.001	>0.99
Antibody titer	20.0 (20.0–77.3)	302.0 (76.5–1210.5)	418.5 (204.5–1008.5)	<0.001	0.50
Delta variant, sVNT50					
Detectable antibody titer ^d^	2 (20.0)	15 (88.2)	30 (93.8)	<0.001	0.60
Antibody titer	20.0 (20.0–31.8)	139.0 (56.5–1058.5)	239.5 (109.8–470.3)	<0.001	0.93
Omicron variant, sVNT50					
Detectable antibody titer ^d^	5 (50.0)	13 (76.5)	29 (90.6)	0.01	0.22
Antibody titer	33.0 (20.0–82.5)	124.0 (47.0–347.5)	154.5 (83.5–451.0)	0.003	0.44

sVNT, SARS-CoV-2 surrogate virus neutralization test. Data are presented as numbers (%) or medians (interquartile range) unless indicated otherwise. ^a^ No vaccination or 1st vaccination before SARS-CoV-2 infection. ^b^ Incomplete vaccination vs. 3rd vaccination before SARS-CoV-2 infection. ^c^ Second vaccination vs. third vaccination before SARS-CoV-2 infection. ^d^ All sera with a 50% reduction in signal at a 1:40 dilution were considered detectable.

**Table 3:** Comparison of serologic results in each cohort that did or did not receive subsequent 4th vaccination and with history of 3rd vaccination before SARS-CoV-2 infection.

Variables	3rd Vaccination (*n* = 32)	Healthcare Worker (*n* = 39)	4th Vaccination after COVID-19 (*n* = 27)	*p* Value ^a^	*p* Value ^b^
Serologic test					
S clade, sVNT50					
Detectable antibody titer ^c^	30 (93.8)	37 (94.9)	27 (100.0)	0.50	0.51
Antibody titer	418.5 (204.5–1008.5)	362.0 (246.0–652.0)	603.0 (383.0–945.0)	0.25	0.03
Delta variant, sVNT50					
Detectable antibody titer ^c^	30 (93.8)	35 (89.7)	27 (100.0)	0.50	0.14
Antibody titer	239.5 (109.8–470.3)	287.0 (103.0–433.0)	405.0 (253.0–753.0)	0.03	0.03
Omicron variant, sVNT50					
Detectable antibody titer ^c^	29 (90.6)	33 (84.6)	24 (88.9)	>0.99	0.73
Antibody titer	154.5 (83.5–451.0)	121.0 (66.0–162.0)	156.0 (74.0–281.0)	0.49	0.17

sVNT, SARS-CoV-2 surrogate virus neutralization test. Data are presented as numbers (%) or medians (interquartile range) unless indicated otherwise. ^a^ Patients who received 3rd dose before SARS-CoV-2 infection and did not receive 4th dose after SARS-CoV-2 infection vs. patients who received 3rd dose before SARS-CoV-2 infection and received 4th dose after SARS-CoV-2 infection. ^b^ Healthcare workers who received 3rd dose before SARS-CoV-2 infection and did not receive 4th dose after SARS-CoV-2 infection vs. patients who received 3rd dose before SARS-CoV-2 infection and received 4th dose after SARS-CoV-2 infection. ^c^ All sera with a 50% reduction in signal at a 1:40 dilution were considered detectable.

## Data Availability

The datasets generated and/or analyzed during the current study are available from the corresponding author upon reasonable request.
